# Computer-aided pattern scoring – A multitarget dataset-driven workflow to predict ligands of orphan targets

**DOI:** 10.1038/s41597-024-03343-8

**Published:** 2024-05-23

**Authors:** Katja Stefan, Vigneshwaran Namasivayam, Sven Marcel Stefan

**Affiliations:** 1 University of Oslo and Oslo University Hospital, Department of Pathology, Rikshospitalet, Sognsvannsveien 20, 0372 Oslo, Norway; 2 University of Lübeck and University Medical Center Schleswig-Holstein, Lübeck Institute of Experimental Dermatology, Medical Systems Biology Division, Medicinal Chemistry and Systems Polypharmacology, Ratzeburger Allee 160, 23538 Lübeck, Germany; 3 University of Bonn, Pharmaceutical Institute, Department of Pharmaceutical and Cellbiological Chemistry, An der Immenburg 4, 53121 Bonn, Germany; 4 Medical University of Lublin, Department of Biopharmacy, Chodzki 4a, 20-093 Lublin, Poland

**Keywords:** Drug discovery and development, Chemical biology

## Abstract

The identification of lead molecules and the exploration of novel pharmacological drug targets are major challenges of medical life sciences today. Genome‐wide association studies, multi-omics, and systems pharmacology steadily reveal new protein networks, extending the known and relevant disease-modifying proteome. Unfortunately, the vast majority of the disease-modifying proteome consists of ‘orphan targets’ of which intrinsic ligands/substrates, (patho)physiological roles, and/or modulators are unknown. Undruggability is a major challenge in drug development today, and medicinal chemistry efforts cannot keep up with hit identification and hit-to-lead optimization studies. New ‘thinking-outside-the-box’ approaches are necessary to identify structurally novel and functionally distinctive ligands for orphan targets. Here we present a unique dataset that includes critical information on the orphan target ABCA1, from which a novel cheminformatic workflow – computer-aided pattern scoring (C@PS) – for the identification of novel ligands was developed. Providing a hit rate of 95.5% and molecules with high potency and molecular-structural diversity, this dataset represents a suitable template for general deorphanization studies.

## Background & Summary

Only 1–2% of the disease-modifying proteome is considered druggable^1^, while all other proteins are orphan targets of which intrinsic ligands/substrates, (patho)physiological roles, and/or modulators are unknown. Particularly small-molecule ligands/substrates/modulators of orphan targets are in need (i) to properly study their physiology and pathology; and (ii) as starting points for hit-to-lead optimization and therapeutic development studies. One prime criterion for industrial investment and production, clinical application, as well as therapy effectiveness is the originality of the drug-to-be, mainly characterized by molecular-structural distinction and innovative modes-of-action. Despite advances in structural biology (*e.g*., cryo-EM, X-ray, STD-NMR, *etc*), providing more and more structural insights even into orphan targets, this information can in most cases not be harnessed for rational drug discovery approaches. Thus, novel strategies are necessary to gain potential drug candidates of the future^2^.

One strategy to get hold of orphan targets is the use of knowledge gained from sibling proteins of which more knowledge has accumulated, termed ‘target repurposing’^2–6^. Generally, proteins of both the same (super-/sub-)family and of phylogenetic distance may contain conserved structural motifs (‘superfolds’^2,7–10^) to which ligands of these well- or less-studied sibling proteins may (partially/mutually) bind (‘supersites’^2,7,11–14^). This may open up a new perspective even for entire under-studied (super-/sub-) families of proteins, and ligands of such orphan protein families, even though of limited potency or binding affinity, could be considered as ‘privileged ligands’^2,14–16^. The acquisition, comprehension, and utilization of molecular-structural coherences between privileged ligands and orphan targets is key, and the compilation of a dataset that enables the set-up of a rational workflow to define and utilize ‘superpatterns’^2^, *i.e*., partial structures and substructural elements that (may) conserve the targeting to supersites of the orphan target-of-interest, as novel scaffolds for subsequent hit-to-lead optimization studies was the aim of the present work.

The ATP-binding cassette transporter ABCA1 is a pharmacologically orphan (= barely druggable) transporter involved in malignant^17–19^, metabolic^19,20^, and neurodegenerative diseases^19,21^. To this date, only 14 inhibitors of human ABCA1 or any other non-human ortholog have been identified. Most inhibitors were very weakly active in the high double-digit to triple-digit micromolar concentration ranges, while others with higher potency have on the one hand strong cytotoxicity, and are on the other hand molecular-structurally indistinctive (*e.g*., cyclosporine A, sirolimus, and tacrolimus)^21^. Thus, ABCA1 belongs to the 90% of the human ABC transporter proteome (‘ABC-ome’) that cannot or only barely be targeted by (weak) small-molecule modulators. ABCA1 belongs in addition to a poorly explored subfamily of ABC transporters for which small-molecule modulators are mostly absent. However, novel lead molecules with molecular-structural distinction could provide medicinal chemistry with the necessary tools to develop novel agents targeting ABCA1, providing real opportunities in, for example, Alzheimer’s disease^21^, atherosclerosis^20^, or melanoma^17^. Thus, we selected ABCA1 as model orphan target to develop a multitarget dataset- and substructural pattern-based workflow as a template framework to address orphan targets and explore them as potential pharmacological targets of the future.

## Methods

**Fig. 1:**
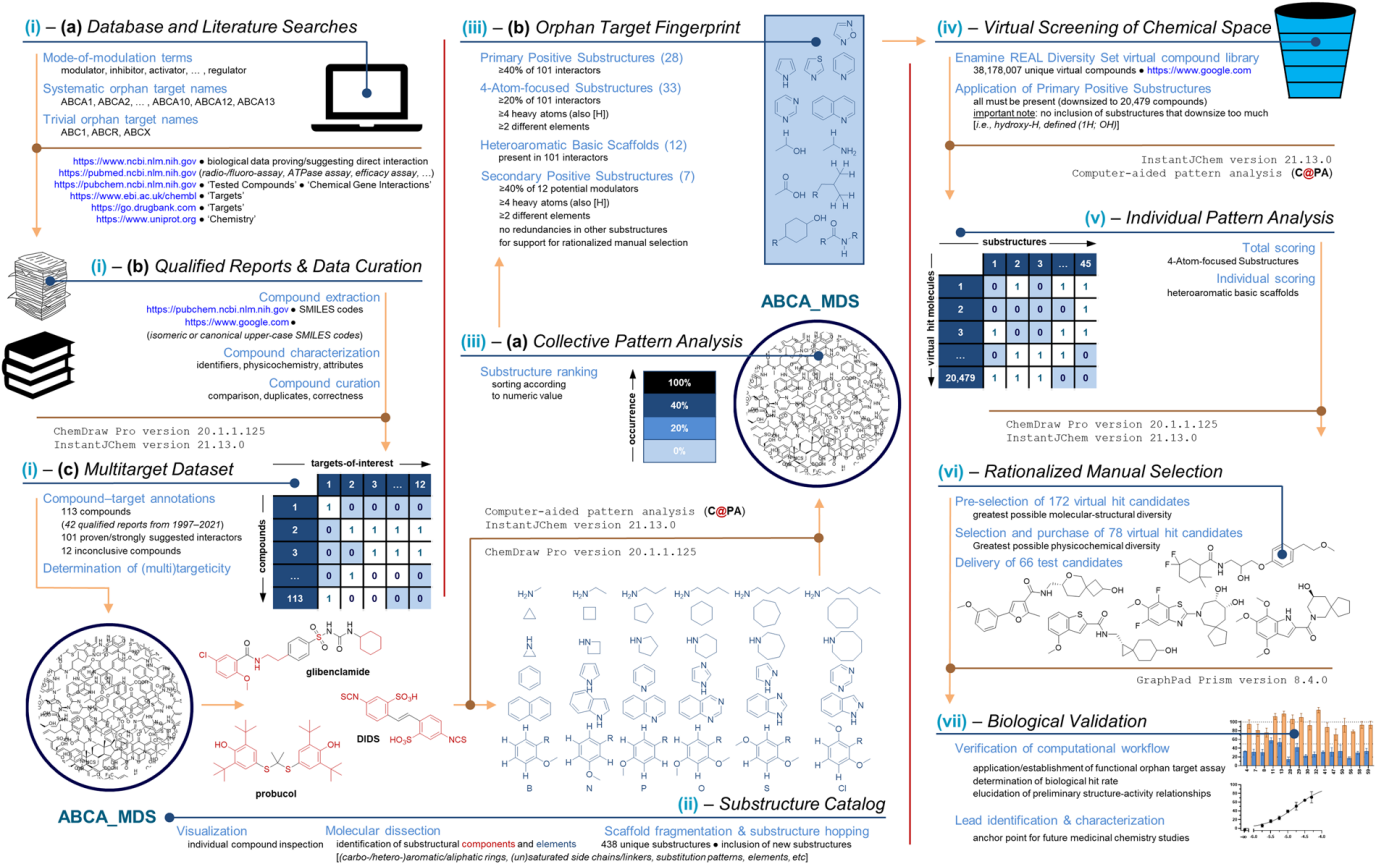
Generalized visualization of the herein presented dataset and associated workflow – computer-aided pattern scoring (C@PS) – to gain novel lead molecules or orphan targets.

The dataset presented in this report constitutes a framework for the intended workflow which was a seven-step process: (i) Database and literature searches (a), data curation (b), and data correlation (*i.e*., molecular-structural landscape of A-subfamily ABC transporter modulators to the target landscape of A-subfamily ABC transporters) to form the multitarget dataset (c). This step was accomplished by the use of various specific search terms and selection criteria; (ii) generation of a substructure catalog based on visualization of the listed A-subfamily ABC transporter modulators as well as our previous reports^22,23^. A heavy atom substitution scheme (Supplementary Fig. S1) was applied to increase the molecular-structural diversity of the substructure catalog^11^; (iii) application of collective pattern analysis^11,24^ elucidating the percentage occurrence of each substructure analyzed within the entire group of A-subfamily ABC transporter modulators. The final results were ranked according to the numeric percentage values, and important substructures were collected for further processes to an ABCA-focused fingerprint according to defined rule schemes as outlined below; (iv) virtual screening of chemical space using the most pronounced substructures resulting in a virtual compound selection; (v) application of individual pattern analysis^25^ by determining the occurrence of a set of pronounced substructures as identified from step (iii). A binary pattern distribution scheme was generated defining the presence (‘1’) or absence (’0’) of the respective substructures as reported earlier^22,23^, and scoring of the virtual compound selection according to these pronounced substructures enabled the extraction of potential hit candidates; (vi) rationalized manual selection based on the statistics as determined in step (v). A set of potential hit candidates was compiled according to a rule scheme as defined below, and selected candidates were obtained according to availability and/or affordability for biological validation; (vii) biological validation by assessing the virtual hit candidates in state-of-the-art *in vitro* test systems^21^. In the following sections, a detailed description of the performed steps can be found that are also summarized and visualized in Fig. 1.

### A-Subfamily ABC Transporters-focused Multitarget Dataset (ABCA_MDS)

#### Database and literature searches

In order to collect the entirety of information on small-molecules that interact with all A-subfamily ABC transporters (termed as ‘interactors’^21^), a deep manual literature search was conducted for qualified reports. The National Center for Biotechnology Information (NCBI; https://www.ncbi.nlm.nih.gov)^26^ was searched for the terms ‘modulator’, ‘modulation’, ‘inhibitor’, ‘inhibition’, ‘activator’, ‘activation’, ‘corrector’, ‘correction’, ‘substrate’, ‘ATPase’, ‘susceptibility’, ‘stabilizer’, ‘stabilization’, ‘regulator’, ‘regulation’, ‘inducer’, ‘induction’, and ‘downregulation’, each in combination with every single A-subfamily ABCA transporter name including common trivial names, *i.e*., ‘ABCA1’, ‘ABC1’, ‘ABCA2’, ‘ABCA3’, ‘ABCA4’, ‘ABCR’, ‘ABCA5’, ‘ABCA6’, ‘ABCA7’, ‘ABCX’, ‘ABCA8’, ‘ABCA9’, ‘ABCA10’, ‘ABCA12’, and ‘ABCA13’, taking both reports about human and non-human orthologs into account.

The found reports were searched for potential interactors and the corresponding biological data that proved or suggested direct interaction with the respective A-subfamily ABC transporter by, *e.g*., modulation of the transport of a (radio-/fluoro-labelled) substrate (measure: radio-/fluoro-intensity distribution between intra- and extra-cellular/-vesicular space after a certain time period), modulation of the ABCA transporter ATPase [measure: colorimetric detection of phosphate (P_i_) production after a certain time period], or increased susceptibility of cancer cell lines expressing (an) A-subfamily ABC transporter(s) by the use of an adjuvant (measure: colorimetric detection of cell viability at exposure to an antineoplastic agent without or with supplemented adjuvant).

The found reports were also searched for relevant literature in the references sections for the extraction of further qualified reports. It shall be taken note that the search did not anticipate the identification and listing of small-molecules that interfered with any kind of regulation (*i.e*., regulators, inducers, and downregulators) of ABCA transporters including the expression on mRNA and protein levels or trafficking, as these processes involved entire pathways that may not be linked to direct interaction with the respective ABCA transporter(s). Nevertheless, such reports were searched for because (i) they may include biological data on small-molecules used to functionally assess the A-subfamily ABC transporter(s)-of-interest; and (ii) the respective references sections of these reports may provide other, relevant reports of such small-molecules. Compounds that increased ‘susceptibility’ of ABCA transporters-expressing cancer cell lines were included for two reasons (i) the used antineoplastic agent could have been a substrate of the ABCA transporter(s)-of-interest (as demonstrated earlier for vesicular ABCA transporters)^27^; and (ii) the sensitizing adjuvant may potentially have interacted directly with the ABCA transporter(s)-of-interest. Given the scarceness of data on ABCA transporters in general, these compounds were also listed.

In addition, other databases were searched for the respective transporter names. PubChem (https://pubchem.ncbi.nlm.nih.gov) was searched in the categories ‘Tested Compounds’ (inclusion of as ‘active’, ‘unspecified’, and ‘inconclusive’ annotated compounds; exclusion of as ‘inactive’ annotated compounds) and ‘Chemical Gene Interactions’ (inclusion of ‘susceptibility’; exclusion of ‘expression’, ‘splicing’, ‘methylation’, ‘phosphorylation’, and ‘oxidation’). ChEMBL (https://www.ebi.ac.uk/chembl) and DrugBank (https://go.drugbank.com) were searched for the respective ‘Targets’, and UniProt (https://www.uniprot.org) was specifically searched for the ‘Chemistry’ section for the identification of further potential small-molecule modulators. Due to data scarceness, most searches resulted in zero returns; however, suggested molecules and associated reports were matched with the NCBI searches, and relevant information was merged.

#### Curation of collected data

All relevant reports were individually checked and qualified small-molecules were listed including their molecular structure conserved as SMILES codes. The information on the SMILES codes were collected from PubChem (https://pubchem.ncbi.nlm.nih.gov) and compared to the respective 2D representation as present in the corresponding report or supplementary information, if applicable. In case a compound could not be retrieved from PubChem, it was searched on Google (https://www.google.com) and relevant 2D representations were drawn from trustworthy web pages [*e.g*., commercial vendors, as for example, Avanti Polar Lipids (https://avantilipids.com), or other open-access tertiary literature) using ChemDraw Pro version 20.1.1.125. Isomeric SMILES were considered where applicable, and all SMILES were listed in the upper-case description scheme as described earlier^23^ and compared to each other to exclude duplicates using InstantJChem version 21.13.0. In total, 113 small-molecules were identified from 42 reports (1997–2021) of which 101 fulfilled the criteria of proven/strongly suggested direct interaction with A-subfamily ABC transporters (Sheet 1, Table A in the dataset), while the other 12 were rather inconclusive and served as support for later stages in the virtual hit compound selection processes (Sheet 1, Table B in the dataset).

### Computer-aided pattern analysis

#### Generation of the substructure catalog of the dataset

In order to develop a chemical pattern-based fingerprint for the prediction of structurally novel modulators of the orphan target-of-interest (*i.e*., ABCA1), a thorough understanding of the molecular-structural landscape of (potential) interactors of this orphan target (*i.e*., ABCA1) and related targets (*i.e*., other A-subfamily ABC transporters) is necessary. We visualized every single molecule of the ABCA_MDS [*e.g*., glibenclamide, 4,4′-Diisothiocyanostilbene-2,2′-disulfonic acid (DIDS), or probucol], and dissected it into its molecular-structural fragments using ChemDraw Pro version 20.1.1.125. These fragments [*e.g*., single-standing/centered/connected/bridged/fused/condensed (carbo-/hetero-)aromatic/aliphatic rings, (un)saturated side chains/linkers, substitution patterns, elements, *etc*] were subsequently individually extended by derivatization applying a heavy atom substitution scheme (Supplementary Fig. S1) as described earlier^11^ (scaffold fragmentation and substructure hopping). The derivatization was necessary to identify fragments that are not easily identifiable at first glance and to increase the quantity of substructural properties, and thus, increase the quality of molecular-structural diversity of the resultant substructure catalog within the dataset. The increased diversity of the substructure catalog will, on the other hand, also lead to an increased diversity of the output molecules after the virtual screening of chemical space. Molecular-structural diversity is key to overcome the molecular-structural limits given by the rather homogenous input compounds of the ABCA_MDS (mostly sterans and/or fatty acids), and enables the identification of molecules of high originality and innovation.

#### Collective pattern analysis

Every identified chemical substructure was searched for in the ABCA_MDS^11,24^ (loaded as.csv file) applying the query search function of InstantJChem version 21.13.0, particularly (i) the entire set of 113 identified ABCA transporter modulators; (ii) the subgroup of 101 small-molecules that were proven or strongly suggested to directly interact with ABCA transporters; and (iii) the 12 modulators that provided rather inconclusive results on ABCA transporter modulation. In total, 438 active (= present) substructures were identified, of which 69 were entirely new and had never been reported before^22,23^. Sheet 2, Table C in the dataset provides the ranked list of active substructures and their calculated percentages (calculation by the quotient of the number of compounds that contained the respective substructure divided by the number of qualified compounds; either 113, 101, or 12). In addition, we included 279 substructures of our previous datasets that were not present within the 113 compounds, which could serve for a negative fingerprint at a later stage of usage of the dataset.

#### Embedding an A-subfamily ABC transporters-focused fingerprint into the dataset

The ranked lists of occurring substructures were analyzed in-depth to extract the relevant substructures for virtual screening of chemical space: (i) the most abundant substructures were extracted that occurred in at least 40% of the 101 proven or strongly suggested direct interactors of A-subfamily ABC transporters (termed ‘Primary Positive Substructures’; in total: 28 substructures; Sheet 3, Table D of the dataset); (ii) many of these substructures were in part very small and molecular-structurally unspecific, *i.e*., their molecular compositions and substitution patterns allowed a high degree of variation (in detail explained in Supplementary Fig. S2). Therefore, we investigated also other substructures that occurred in at least 20% of the molecules of the ABCA_MDS, however, which had at least four heavy atoms (defined H-atoms represented as [H] in SMILES codes also counted; [R] did not count; at least two different elements). This measure increased not only the molecular-structural diversity of the output fingerprint but promoted also larger substructures that have generally a lower likelihood of being further up in the ranking due to their higher specificity (Supplementary Fig. S2). Particularly the promotion of defined ( = irreplaceable) H-atoms strengthens the polypharmacology of the output fingerprint and the chance to identify ABCA1 modulators by compounds bearing substructures of templates that did not necessarily target ABCA1^11,23–25^. In total, 33 such ‘4-Atom-focused Substructures’ were identified (Sheet 3, Table E of the dataset); (iii) the identification of novel core structural features and basic scaffolds are critical for hit-to-lead identification processes from a medicinal-chemical point-of-view. Thus, we searched the substructures for heteroaromatic scaffolds independent from the absolute or relative number/percentage of compounds of the ABCA_MDS in which they were present. Here, we identified 12 scaffolds (Sheet 3, Table F of the dataset); (iv) finally, the 12 compounds that did not demonstrate immediate interaction with A-subfamily ABC transporters or did only weakly suggest direct interaction were separately analyzed for the substructures present in at least 40% ( = 5 compounds) of these molecules using the same 4-Atom-scheme as described in (ii). Sheet 3, Table G of the dataset outlines these 7 ‘Secondary Positive Substructures’ (no redundancies to Tables D–G). These were not used for virtual screening purposes, however, supported necessary manual selection steps for the best possible outcome.

#### Dataset-guided virtual screening of chemical space for potential orphan target modulators

In the next step, the elucidated fingerprint consisting of four individual parts was translated into a virtual screening protocol. We used the *REAL* Diversity Set compound library from Enamine (https://enamine.net) consisting of 38,178,007 unique compounds. These compounds were screened for the 28 Primary Positive Substructures, which needed to be present in all returned virtual hit molecules. This step led to a reduction to 20,479 molecules. Importantly, it needs to be taken note that the substructure ‘hydroxy-H, defined (1H; OH)’ (Substructure Identifier 0044) has not been applied as it downsized the compound library to 7,798 molecules only, which did not leave enough space for further processing.

#### Individual pattern analysis and binary pattern distribution scheme in the dataset

In the next step, a binary pattern distribution scheme was generated as described earlier^22,23^ applying the query search function of InstantJChem version 21.13.0, searching the 33 4-Atom-focused Substructures as well as the 12 Basic Scaffolds against the 20,479 potential ABCA1 interactors^23,25^. Of importance, the search anticipated only the presence ( = quality)^22,23^ of the searched substructure but not number of appearances ( = quantity)^23^. The scheme, which can be found in Sheet 4, Table H of the dataset, was used for the rationalized drug selection process. Importantly, the entire dataset in general and these 20,489 compounds in particular may serve as source for modulators of either other ABCA transporters, or other cholesterol/phospholipid/bile acid/fatty acid transporters of the ABC (*e.g*., ABCB11, ABCG1, and ABCG4) or solute carrier (SLC) superfamilies [*e.g*., NTCP (*SLC10A1*), FATP1 (*SLC27A1*), and NPC1 (*SLC65A1*)].

#### Rational compound selection for modulators of orphan targets by pattern scoring

The binary pattern distribution scheme was used to rationally pre-select for final examination and purchase. The aim of the study was (i) to identify novel modulators of ABCA1, and (ii) to promote molecular-structural diversity of identified potential modulators. The strategy for compound pre-selection was as follows: (i) selection of the top compounds with most 4-Atom-focussed Substructures (27–25; number of compounds: 24; Sheet 5, Table I of the dataset); (ii) 1,2,5-Oxadiazoles with most 4-Atom-focused Substructures (19–15; number of compounds: 5; Sheet 5, Table J of the dataset); (iii) Pyrroles (no indoles) with most 4-Atom-focused Substructures (22–16; numbers of compounds: 17; Sheet 5, Table K of the dataset); (iv) Pyridines (no quinolines, but with isoquinolines) with most 4-Atom-focused Substructures (23–21; number of compounds: 11; Sheet 5, Table L of the dataset); (v) Thiazoles (including benzothiazoles) with most 4-Atom-focused Substructures (21–19; number of compounds: 13; Sheet 5, Table M of the dataset); (vi) Pyrimidines (no quinazolines) with most 4-Atom-focussed Substructures (21–19; number of compounds: 11; Sheet 5, Table N of the dataset); Quinolines with most 4-Atom-focused Substructures (21–20; number of compounds: 12; Sheet 5, Table O of the dataset); (vii) Thiophenes (including benzothiophenes) with most 4-Atom-focused Substructures (22–18; number of compounds: 15; Sheet 5, Table P of the dataset); (viii) Indoles with most 4-Atom-focussed Substructures (22–20; number of compounds: 21; Sheet 5, Table Q of the dataset); (ix) Furans (no benzofurans) with most 4-Atom-focused Substructures (21–17; number of compounds: 14; Sheet 5, Table R of the dataset); (x) pyridyl-cations (not available in the dataset); (xi) Quinazolines (no quinazolinones) with most 4-Atom-focuses Substructures (20–16; number of compounds: 15; Sheet 5, Table S of the dataset); and (xii) benzofurans with most 4-Atom-focused Substructures (21–19; number of compounds: 14; Sheet 5, Table T of the dataset).

In total, 172 compounds of broadest possible molecular-structural diversity were pre-selected, from which a final selection of 92 compounds was chosen for purchase after visualization and under consideration of Secondary Positive Substructures (Sheet 3, Table G of the dataset) as well as physicochemical properties calculated octanol-water partition coefficient (CLogP), molecular weight (MW), molar refractivity (MR), and topological polar surface area (TPSA)^28^. Sixty-six of these 90 compounds (compounds **1**–**66**; Sheet 6, Table U of the dataset) could be delivered, while 24 compounds (Sheet 6, Table V of the dataset) were unavailable. Again, these within the dataset filed, selected, as well as molecular-structurally and physicochemically highly diverse compounds may also represent the best starting points to explore either other ABCA transporters or other lipid transporters of the ABC or SLC transporter superfamilies.

## Data Records

The dataset is available open-access in an .xlsx format under http://www.zenodo.org^29^ as well as the http://www.panabc.info web page, and its use is free of charge. The dataset consists of:

(i) the ABCA_MDS with 113 (potential) A-subfamily ABC transporter(s) interactors including (a) their individual ABCA_MDS_ID, (b) original names according to the original report(s), (c) common abbreviations, (d) synonyms if applicable, (e) the systematic IUPAC name determined using ChemDraw Pro version 20.1.1.125, (f) SMILES codes as obtained from public databases and/or public information resources, (g) PubChem ID, (h) ChEMBL ID, (i) DrugBank Accession Number, (j) IUPHAR/BPS Guide to Pharmacology Ligand ID, (k) Chemical Abstract Service (CAS) Number, (l) calculated octanol-water partition coefficient (CLogP), (m) calculated solubililty (CLogS), (n) molecular weight (MW), (o) molar refractivity (MR), (p) topological polar surface area (TPSA; all physicochemical properties calculated using MOE version 2019.01), (q) number of hydrogen (H-) bond donors, (r) number of H-bond acceptors, (s) number of rotatable bonds, (t) number of heavy atoms, (u) name of addressed target(s), (v) UniProt Protein ID, (w) PubChem Protein ID, (x) ChEMBL Target ID, (y) IUPHAR/BPS Guide to Pharmacology Target ID, and (z) an exemplary literature reference [PubMed ID (PMID), https://pubmed.ncbi.nlm.nih.gov], all visualized in Sheet 1);
(ii) substructure catalog of 438 active and 279 inactive ( = not present)^22,23^ substructures (total: 717 substructures) generated applying ChemDraw Pro version 20.1.1.125 and InstantJChem version 21.13.0, including (a) substructure identifier under consideration of the nomenclature of our previous reports^22,23^, (b) substructure name, (c) SMILES code, (d) statistics related to all 113 ABCA_MDS compounds, (e) statistics related to the 101 proven/strongly suggested ABCA_MDS compounds, and (f) statistics related to the 12 inconclusive A-subfamily ABC transporter(s) interactors (Sheet 2). The substructures are sorted according to abundance in the 113 compounds of the ABCA_MDS (top–bottom);
(iii) extracted, relevant substructures for virtual screening of chemical space, including (a) Primary Positive Substructures, (b) 4-Atom-focused Substructures, (c) Basic Scaffold Substructures, and (d) Secondary Positive Substructures (Sheet 3);
(iv) binary pattern distribution scheme of 20,479 virtual hit candidates, including (a) SMILES code, (b) Enamine ID, (c) substructure identifier of relevant substructure, (d) number of compounds containing particular relevant substructure, (e) number of heavy atoms per relevant substructure, (f) number of defined ( = irreplaceable) hydrogens per relevant substructure, (g) SMILES code of each relevant substructure, (h) name of each relevant substructure, (i) and binary code consisting of present ( = active; ‘1’) and absent ( = inactive; ‘0’) relevant substructures (Sheet 4);
(v) pre-selected virtual hit candidates from different groups, particularly (a) top-ranked 4-Atom-focused Substructure compounds, (b) 1,2,5-Oxadiazoles, (c) Pyrroles, (d) Pyridines, (e) Thiazoles, (f) Pyrimidines, (g) Quinolines, (h) Thiophenes, (i) Indoles, (j) Furans, (k) Quinazolines, and (l) Benzofurans (Sheet 5). Each compound is annotated with its (a) SMILES code, (b) number of 4-Atom-focused Substructures, (c) CLogP, (d) CLogS, (e) MW, (f) MR, (g) TPSA, (h) number of H-bond donors, (i) number of H-bond acceptors, (j) number of rotatable bonds, and (k) number of heavy atoms; and
(vi) selected and ordered virtual hit candidates for biological validation, including (a) available compounds that were eventually delivered, and (b) compounds that could not be delivered (Sheet 6).

All Tables A–V in Sheets 1–6 of the dataset are sorted according to alphabet or occurrence [Table A–B: alphabetic sorting of 113 ABCA_MDS compounds (top–bottom); Table C: active substructures sorted according their occurrence in 113 ABCA_MDS compounds (top–bottom); Table D–G: Primary, 4-Atom-focused, Basic Scaffold, and Secondary Positive Substructures sorted according to their occurrence in 113 ABCA_MDS (top–bottom); Table H: binary pattern distribution scheme of 4-Atom-focused and Basic Scaffold Substructures sorted according to occurrence (left–right) and the 20,479 potential virtual hit candidates according to occurrence of 4-Atom-focused substructures (top–bottom); Tables I–T: 176 pre-selected virtual hit candidates sorted according to the occurrence of the respective substructures/basic scaffolds (top–bottom); and Tables U–V: selected virtual hit candidates sorted according to deliverability].

## Technical Validation

### Validation of input data of the dataset – molecular-structural aspects

The 113 compounds of the ABCA_MDS were loaded as either canonical or isomeric SMILES codes using the MarvinSketch editor implemented in InstantJChem version 21.13.0 and compared to the 2D representation of the original reports and/or public databases [PubChem (https://pubchem.ncbi.nlm.nih.gov), ChEMBL (https://www.ebi.ac.uk/chembl), DrugBank (https://go.drugbank.com), IUPHAR/BPS Guide to Pharmacology (https://www.uniprot.org), and CAS Common Chemistry (https://commonchemistry.cas.org). If the used SMILES code appeared as intended without any error, it was considered valid. The same holds true for the SMILES codes of the substructure catalog, which were rigorously checked for chemical integrity, duplicity, and annotation to previous labels^22,23^ (*i.e*., identifiers) for better visibility according to FAIR principles and for less confusion by multiple identifiers. Of note, pattern analysis is ‘blind’ for stereoisomers, and thus, isomeric SMILES codes behave in pattern analysis as canonical SMILES codes.

### Validation of input data of the dataset – bioactivity aspects

In total, only 5 compounds of the ABCA_MDS were annotated to half-maximal inhibition concentrations against the orphan target-of-interest, ABCA1, *i.e*., cyclosporine A, pimecrolimus, sirolimus, tacrolimus, and valspodar^21^, while all other bioactivity values were given only in single-concentration measurements in which the observed biological effects were rather weak in the double- to triple-digit concentration ranges. As only 21 A-subfamily ABC inhibitors (ABCA1: 14; ABCA8: 7) are known^21^, and only 5 of these compounds were associated to IC_50_ values^21^, these numeric values could not be taken into account in the computational model to develop novel interacting molecules. On the other hand, this workflow was particularly developed to explore orphan targets in which generally (almost) no structural, functional, or molecular information is available, and thus, this obstacle was considered as rather uncritical. Most compounds interacted with ABCA1 (51 compounds), followed by ABCA4 (26 compounds), ABCA3 (22 compounds), ABCA8 (12 compounds), ABCA7 (7 compounds), and ABCA2 (8 compounds). In total, 103 compounds had one target only, while 7 compounds addressed 2 targets, and 3 compounds targeted 3 A-subfamily ABC transporters. The entire dataset was designed to enable multidimensional expansion, for example, to chart the growing bioactivity or targeticity landscapes.

### Validation of input data of the dataset – physicochemical aspects

**Fig. 2:**
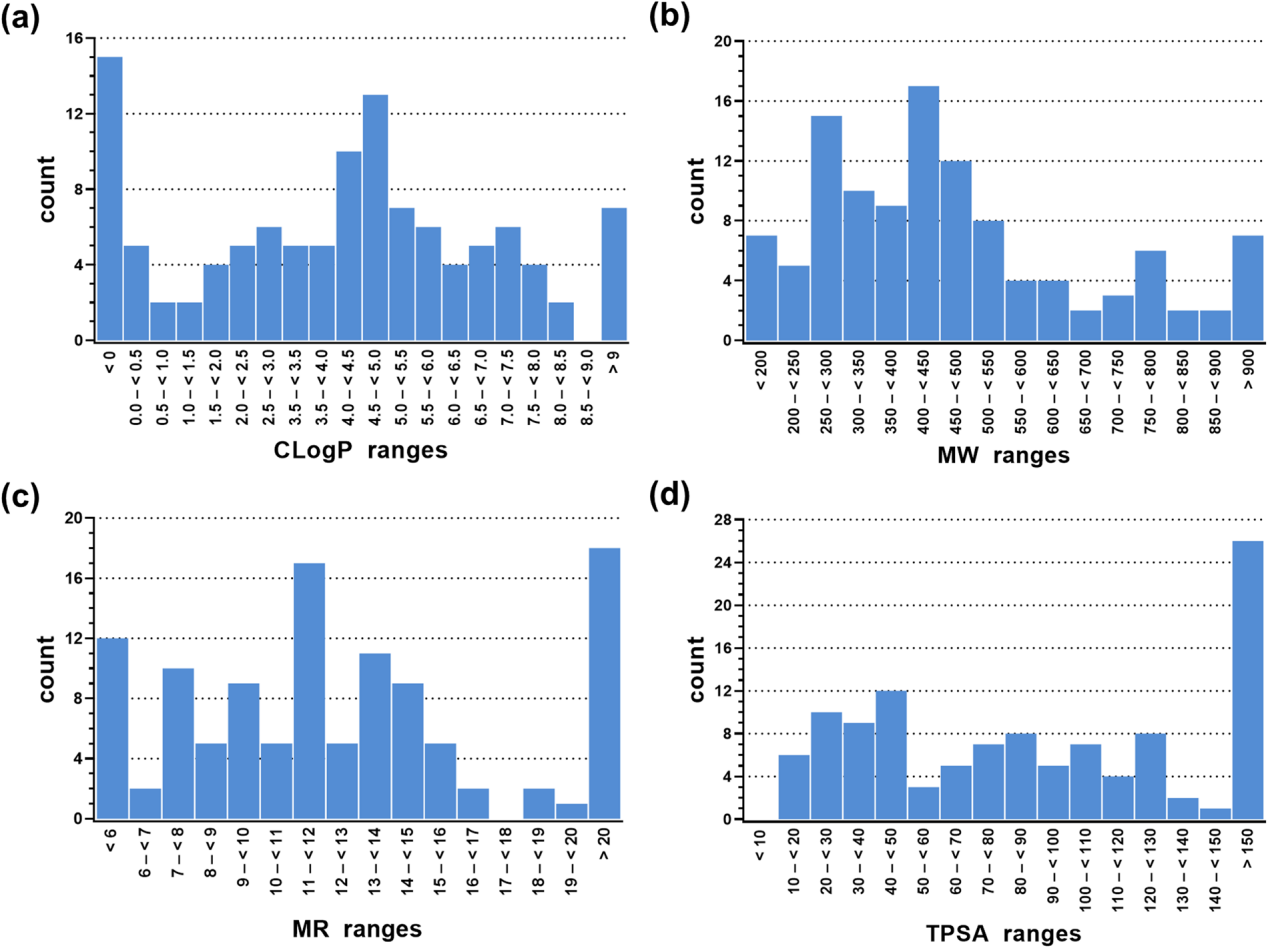
Analyses of the 113 compounds of the ABCA_MDS for the physicochemical properties CLogP (**a**), MW (**b**), MR (**c**), and TPSA (**d**) as determined by MOE version 2019.01.

Almost no molecular coherences for ABCA1 modulation in particular and A-subfamily ABC transporter modulation in general are known. Thus, we anticipated the greatest possible diversity with respect to the physicochemical properties CLogP, MW, MR, and TPSA of the virtually screened compounds to increase the chances of biological hit identification. Physicochemical properties are generally important, not only with respect to ABC transporter modulation^30–33^ but also as descriptors and discriminators for other virtual screening approaches^11,24,25,34^. The ABCA_MDS was assessed with respect to the above mentioned physicochemical properties and compared to previously published datasets^22,23^ for the quality of distribution balance of the numeric values using MOE version 2019.01. Figure 2 shows the obtained results, and Table 1 provides the numeric values for these analyses.

**Table 1:** Statistical survey of median and mean values of the physicochemical properties CLogP, MW, MR, and TPSA amongst relevant groups of compounds of the ABCA_MDS as determined by MOE version 2019.01.

ABCA_MDS compounds	CLogP median	CLogP mean	MW median	MW mean	MR median	MR mean	TPSA median	TPSA mean
Group of 113	4.38	3.90	430.72	479.63	11.93	12.94	84.86	103.94
Group of 101	4.57	4.08	430.72	481.69	11.97	13.09	83.92	102.12
Group of 12	2.70	2.38	423.46	462.27	10.85	11.73	102.07	119.23

Although a (sometimes rather flat) Gaussian distribution could be observed for the assessed physicochemical properties, it was quite noticeable that particularly the extremes of the scale were most pronounced, *i.e*., very low ( = negative) CLogP values, high molecular weight values, high molar refractivity values, and very high TPSA values. These observations can be explained by the molecular composition of some compounds, containing free acids, hydroxy groups, sugar residues, or charged hetero-atoms. Another explanation is the size of the dataset: as the ABCA_MDS contained 113 compounds only, compounds with rather exceptional physicochemical characteristics in the entire small-molecule landscape may impact the overall composition and average values more than in larger datasets. This is supported when looking at two other datasets, the over 1,167 compounds-containing ABC_BPMDS^23^, and the 429 compounds-containing HD_BPMDS^22^. While the former showed almost perfect Gaussian distributions with no extreme compounds, the latter had a significant but low number of compounds at the very edges of the scales. The 113 compounds-containing ABCA_MDS fits perfectly into this correlation.

Physicochemistry is also important with respect to the ADME ( = absorption, distribution, metabolization, elimination) of drugs, and the concept of drug-likeness is known for almost a quarter century now^35^. Considering the physicochemistry of the ‘Lipinski-rule-of-five’, 63.7% and 66.4% of the ABCA_MDS had a CLogP ≤ 5 and MW ≤ 500, respectively. This means that this dataset contains a sufficient portion of suitable candidates for drug discovery purposes, but at the same time, leaves enough physicochemical space beyond these specific attributes to counteract the homogeneity of datasets. On the other hand, 33.6% and 36.3%, respectively (~1/3 of the ABCA_MDS), fell clearly out of the ‘Lipinsky-rule-of-five’, demonstrating the special standing of A-subfamily ABC transporters amongst the target landscape of druggable or to-be-drugged targets – and the special standing of their (potential) interactors in the global small-molecule landscape.

### Validation of input data of the dataset – molecular-structural aspects

**Fig. 3:**
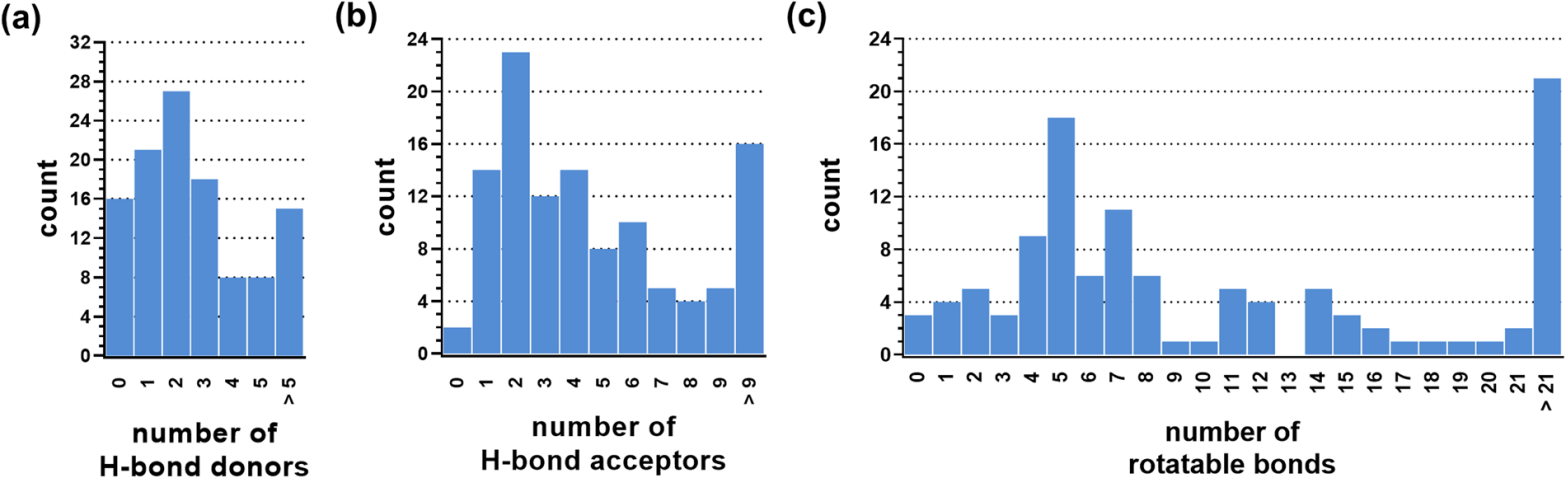
Analyses of 113 compounds of the ABCA_MDS for the numbers of H-bond donors (**a**), H-bond acceptors (**b**), and rotatable bonds (**c**) as determined by MOE version 2019.01.

Similar to physicochemical properties, comprehensive analyses regarding H-bond interactions and molecular flexibility have been conducted neither with respect to ABCA1 nor regarding other A-subfamily ABC transporters despite crucial importance. Thus, we analyzed the ABCA_MDS for the numbers of H-bond donors and acceptors, as well as rotatable bonds. Figure 3 shows the obtained results and Table 2 provides the numeric values.

**Table 2:** Statistical survey of median and mean values of H-bond donors, H-bond acceptors, and rotatable bonds amongst relevant groups of compounds of the ABCA_MDS as determined by MOE version 2019.01.

ABCA_MDS Compounds	H-bond donors median	H-bond donors mean	H-Bond acceptors median	H-bond acceptors mean	rotatable bonds median	rotatable bonds mean
Group of 113	2	2.81	4	5.18	7	9.76
Group of 101	2	2.76	4	5.12	7	10.08
Group of 12	3	3.12	5	5.67	8	7.08

As already observed for the physicochemical properties, Gaussian distributions could be observed for all analyzed attributes, however, particularly extreme compounds with (ultra-)large numbers of H-bond donors and acceptors, but also rotatable bonds were very pronounced. These observations are mostly attributed to polypeptides (*e.g*., cyclosporine A, leukotriene C4, or sirolimus), glycosides (*e.g*., cyclodextrin, digitonin, or phosphatidyl inositol), but also fatty acids-containing compounds (*e.g*., ceramide, phosphatidyl choline, or sphingomyelin).

Concerning drug likeness^35^ and the molecular-structural aspects of the ‘Lipinski-rule-of-five’, 86.7% and 89.4% of the ABCA_MDS compounds had a number of H-bond donors ≤5 and a number of H-bond acceptors ≤10, respectively, outlining the input data as sufficiently drug-like for drug development purposes without the burden of homogeneity. It shall be taken note that potential impairments of prediction capability due to the potential lack of drug-likeness of the input compounds was counteracted by using the *REAL* Diversity Set compound library from Enamine (https://enamine.net) which contains solely drug-like molecules. However, the dataset is entirely compatible with other compound libraries of distinct physicochemical, molecular-structural, and/or pharmacological foci.

Finally, the substructure catalog of in total 438 substructures was used to entirely describe the ABC_MDS from a molecular-structural point-of-view. This is a comparable size to other datasets^22,23^, and thus, we considered it suitable for the subsequent computational analyses. The inclusion of 68 novel substructures also contributed to the originality and innovation of the output molecules. Furthermore, we included 279 inactive substructures from our previous studies^22,23^ that may serve as a negative fingerprint in future studies.

### Validation of output data of the dataset – biological investigation

The purpose of the herein described dataset was the generation and complete description of a workflow to gain novel molecules addressing an orphan target-of-interest. In total, 66 molecules were finally delivered by Enamine which were obtained through a rigorous selection process. These 66 molecules were suggested to be interactors of ABCA1, an orphan transporter of which to this date only 14 inhibitors are known^21^. In order to validate the prediction capabilities of the presented workflow, these 66 molecules were assessed in one of the most commonly used assessment platforms for ABCA1 function^21^, namely a 25-NBD-cholesterol assay and ABCA1-expressing murine J774A.1 macrophages [TB-67, American Type Culture Collection (ATCC); cultured in Dulbecco’s modified eagle medium (DMEM; Biowest, Nuaillé, France)]. In short, 25-NBD-cholesterol [Avanti Polar Lipids (Alabaster, AL, USA); final concentration: 1 µM] is a fluoro-labelled (NBD = *N*-[(7-nitro-2-1,3-benzoxadiazol-4-yl)methyl]amino] derivative of the ABCA1 substrate cholesterol. 25-NBD-cholesterol (20 µL) accumulates in J774A.1 cells [160 µL; 45,000 cells/well; clear flat-bottom 96-well plates (Brand, Wertheim, Germany)] within the cellular membranes in the incubation time of 48 h and becomes extruded by ABCA1. ABCA1 inhibition by addition of the test compounds (20 µL; 24 h prior to addition of 25-NBD-cholesterol) leads to reduced cholesterol efflux, and thus, increased intracellular fluorescence that is detected using an Attune NxT flow cytometer (Invitrogen, Waltham, MA, USA; excitation: 488 nm; emission: 530/30 nm).

**Fig. 4:**
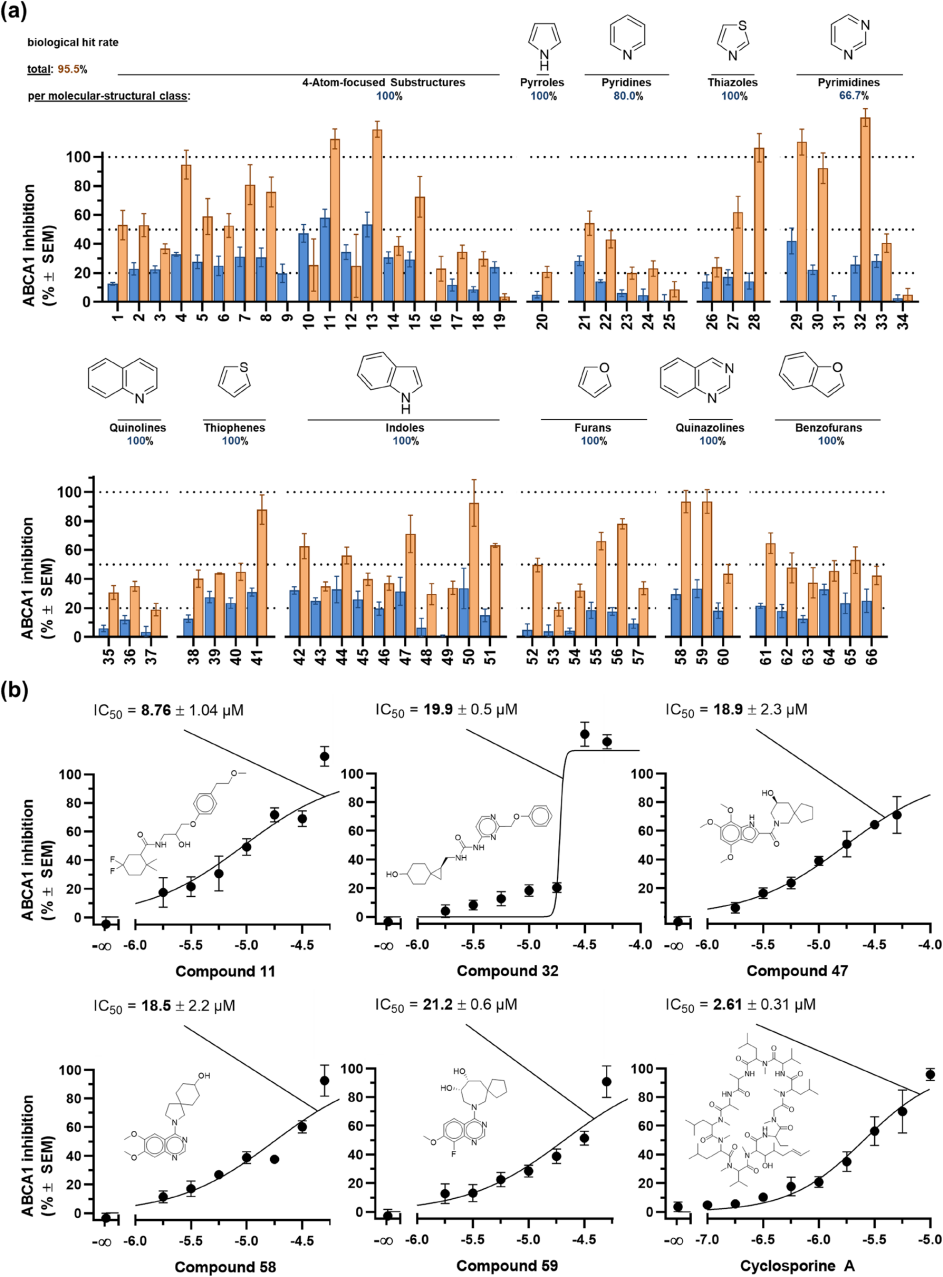
Biological assessment of 66 virtual hit candidates in an initial screening (**a**) at 10 µM (blue) and 50 µM (orange) compound concentrations compared to the effect values of pure cell culture medium (0%) and the reference ABCA1 inhibitor cyclosporine A (100%). The molecules are classified into their molecular-structural classes as specified in the dataset (4-Atom-focused Substructure compounds, Pyrroles, Pyridines, Thiazoles, Pyrimidines, Quinolines, Thiophenes, Indoles, Furans, Quinazolines, and Benzofurans), and the overall hit rate (95.5%) as well as the hit rate for each molecular-structural class (66.7–100%) are given. Shown are mean ± SEM values of at least three independent experiments. (**b**) Concentration-effect curves of compounds **11,** **32,** **47,** **58**, and **59** compared to the reference ABCA1 inhibitor cyclosporine A. Data obtained applying a 25-NBD-cholesterol assay and ABCA1-expressing J774A.1 cells. Shown are mean ± SEM values of at least three independent experiments.

Figure 4(a) provides the screening results. Considering a threshold of 20% inhibition [ + standard error of the mean (SEM)], the herein presented workflow predicted ABCA1 modulators with a total biological hit rate of stunning 95.5% (10 µM only: 62.1%; 50 µM only: 92.4%). These findings are in many ways exceptional, which we would like to point out in the following:

(i) the observed hit rates are extraordinary, even for chemical pattern-based approaches which resulted already in above-average hit rates in the past^11,24,25^. This becomes even more surprising as less than half of the data (45.1%) taken into account to develop the computational model was acquired at the actual orphan target-of-interest, *i.e*., ABCA1. As stated above, only 14 inhibitors of ABCA1 have been described so far^21^, with only 5 of these compounds described with an actual IC_50_ value^21^;
(ii) even a hit rate at 10 µM compound concentration of 62.1%, which is significantly lower than the hit rate at 50 µM compound concentration (92.4%) or the total hit rate (95.5%), is truly exceptional and surprising. Generally, inhibitors of ABCA1 exert their inhibitory activity at much lower, mostly high double- to triple-digit micromolar concentration ranges (apart from very few exceptions, *e.g*., cyclosporine A)^21^. Table 3 summarizes estimated IC_50_ values taking the two test concentrations of 10 µM and 50 µM into account, showing that the estimated IC_50_ values of active compounds were all below 200 µM, and almost half (49.2%) of these compounds had IC_50_ values of <50 µM, which can be considered as potent with respect to ABCA1. These activities are unexpected, as many compounds with much lower biological activity (at in part different targets) were taken into account for model development;

**Table 3:** Estimated IC_50_ values of the 66 assessed virtual hit candidates applying a 25-NBD-cholesterol assay and ABCA1-expressing J774A.1 cells.

Compound	IC_50 (est.)_	Compound	IC_50 (est.)_	Compound	IC_50 (est.)_	Compound	IC_50 (est.)_
*4-Atom-focused*		*Pyrroles*		*Quinolines*		*Furans*	
1	51.4 ± 7.6	20	188 ± 20	35	121 ± 16	51	61.3 ± 5.3
2	42.0 ± 6.7			36	92.5 ± 7.9	52	207 ± 32
3	71.2 ± 6.2	*Pyridines*		37	212 ± 27	53	123 ± 13
4	13.4 ± 1.1	21	37.9 ± 6.6			54	28.5 ± 5.0
5	33.5 ± 7.5	22	68.3 ± 9.1	*Thiophenes*		55	23.4 ± 1.1
6	40.5 ± 8.2	23	194 ± 25	38	66.3 ± 6.5	56	98.0 ± 12.2
7	18.7 ± 4.5	24	168 ± 29	39	50.0 ± 1.8		
8	20.2 ± 3.9	25	*n.i*.	40	54.6 ± 6.3	*Quinazolines*	
9	46.2 ± 11.1			41	17.1 ± 1.8	58	12.6 ± 3.1
10	11.3 ± 1.5	*Thiazoles*				59	9.12 ± 2.9
11	5.08 ± 0.62	26	115	*Indoles*		60	62.2 ± 8.7
12	19.6 ± 2.5	27	26.6 ± 7.9	42	29.4 ± 4.6		
13	5.34 ± 0.71	28	15.3 ± 1.4	43	73.4 ± 6.2	*Benzofurans*	
14	58.2 ± 10.1			44	29.6 ± 5.5	61	33.1 ± 3.4
15	23.4 ± 5.2	*Pyrimidines*		45	59.6 ± 8.4	62	59.6 ± 13.2
16	174 ± 40	29	9.74 ± 2.17	46	74.3 ± 10.3	63	87.4 ± 15.2
17	93.9 ± 9.9	30	20.4 ± 2.8	47	21.5 ± 3.4	64	49.5 ± 11.0
18	122 ± 17	31	*n.i*.	48	127 ± 26	65	41.4 ± 8.0
19	33.9 ± 4.8	32	9.89 ± 0.40	49	117 ± 6	66	60.7 ± 12.4
		33	58.2 ± 9.5	50	13.3 ± 1.5		
		34	*n.i*.	51	35.5 ± 1.9		

IC_50_ values were determined using the two effect-values at 10 µM and 50 µM as well as 0% and 100% values defined by cell culture medium and the ABCA1 reference inhibitor cyclosporine A, respectively. Calculations were performed using GraphPad Prism version 8.4.0 choosing the three-parameter logistic equation; *n.i*. = no inhibition.
(iii) generally, one would not expect hits in the high activity range from a screening, however, 5 compounds indicated activities in the single-digit micromolar concentration range, highlighting them as some of the most potent ABCA1 inhibitors ever reported;
(iv) a high hit rate for the 4-Atom-focused Substructure top rank compounds (100%) could be expected as these compounds maximally conserved the molecular-structural information present in the ABCA_MDS. However, similar hit rates could also be observed for other molecular-structural classes, which is a major finding of this study. While here still the top rank compounds in terms of the 4-Atom-focused substructures were used, the absolute number of these substructures was on average much lower (Pyrroles: 16.8; Pyridines: 21.5; Thiazoles: 19.8; Pyrimidines: 19.5; Quinolines: 20.3; Thiophenes: 19.4; Indoles: 20.6; Furans: 17.9; Quinazolines: 17.0; Benzofurans: 19.8) compared to the overall top ranked compounds (25.4). This indicates a strong robustness of the workflow and the entire dataset it was based upon, which apparently tolerates also a certain degree of substructures not or not knowingly associated with the desired biological effect-of-interest (*e.g*., ABCA1 inhibition). This also means that the pattern-based workflow is suitable for design-in^36–38^ (or design-out^36,37,39^) approaches to shape the (poly)pharmacological profile of future drugs.

In order to validate the biological findings, 5 molecules indicated as potent hits from different molecular-structural classes were tested in full-blown concentration-effect curves. Figure 4(b) shows that all assessed compounds showed concentration-dependency and confirmed their comparable high inhibitory power against ABCA1.

### Validation of output data of the dataset – general specificity and selectivity of the output molecules

Apart from the high hit rate, potency, and structural diversity of the output molecules, it is important to note that the multitarget dataset cross-annotated the molecular-structural and physicochemical information of ligands on a multitude of targets. In order to assess its ABCA-specificity, we tested the hit compounds **4, 7, 8, 11, 13, 28, 29, 30, 32, 41, 46, 47, 50, 58**, and **59** against ABC transporters of three different subfamilies, namely ABCB1, ABCC1, and ABCG2^40^. As can be seen in Supplementary Figure S3, particularly the top compounds with most 4-Atom-focussed Substructures (*i.e*., **4,** **7,** **8,** **11**, and **13**), which adhered most to the ABCA-focused fingerprint, showed least inhibition of other ABC transporters. Compounds containing basic scaffolds, particularly pyrimidine (**29,** **30**, and **32**) and quinazoline (**58** and **59**), showed significant inhibition against either two or three of the assessed transporters, which could be expected given the results of our previous work^11,24,25^. On the other hand, it is unlikely that the compounds bear selectivity toward ABCA1, which can be seen as opportunity to explore other ABCA transporters as well, and warrants future investigations.

## Usage Notes

### Dataset and content

The provided dataset contains valuable information for researchers working in various fields:

(i) the entirety of information known to this date about modulators of ABCA transporters can be found within one single file. Thus, the dataset supports researchers and groups new in the fields of endocrinology^19,20^, neurology^19,21^, oncology^17–19^, and general pathology, but also medicinal chemistry as well as (bio- and chem)informatics;
(ii) all currently known 717 patterns released within this work and our previous reports^22,23^ are summarized within one dataset, facilitating and promoting the application of chemical pattern-based approaches;
(iii) the dataset is split into a number of individual sheets, each sub-divided into information units that enable adherence to or adaption of the described workflow. As over 97% of the disease-modifying proteome is considered undruggable^1^, the dataset offers a framework as cutting-edge tool for the exploration of the unknown.

### Pattern-based workflow

Together with the dataset, this report provides a detailed protocol and description of the pattern scoring-based workflow. In order to apply this workflow toward other orphan targets, the following 7 steps need compliance:

(i) deep literature search under consideration of search terms specific for the orphan target-of-interest and definition of qualification criteria under which qualified reports and qualified small-molecules are identified and filed for further processing;
(ii) generation of an adapted substructure catalog taking filed molecules from step (i) into account. This step is, however, optional as enough substructural patterns are available (in total 717 from three reports^22,23,29^);
(iii) application of collective pattern analysis to obtain necessary statistics. A rule-scheme should be implemented according to which (Primary/Secondary) Positive Substructures are defined and applied for subsequent virtual screening purposes. Depending on the nature of the performed study, one may want to also search for Basic Scaffolds;
(iv) virtual screening of chemical space for molecular-structurally novel molecules of high originality. Many vendors are available providing different(ly focused) virtual compound libraries [*e.g*., ChemDiv (https://www.chemdiv.com), ChemSpace (https://chem-space.com), Enamine (https://enamine.net), MolPort (https://www.molport.com), ZINC (https://zinc20.docking.org), *etc*];
(v) generation of a binary pattern distribution scheme to allow for an entirely rational compound selection. The herein presented workflow for the development of novel ABCA1-targeting agents demonstrated the superiority of a diversity-based strategy which may be wanted to be considered in other fields depending on the orphanization state of the target-of-interest;
(vi) rationalized manual selection under consideration of as many pre-defined rules as possible. Manual selection is, although of high value to acknowledge the empirical experience of researchers, considered as a weak point of virtual screening-based approaches and regularly criticized in peer review processes. One major strength of the herein presented pattern-based workflow was the high degree of rationalization, which finally led to major success;
(vii) biological validation of the new compounds. This step can be the hardest as for most orphan targets – unlike ABCA1 – no functional assays are available. However, cell-biological research has provided the scientific communities with many template functional assays that could be considered, for example, determination of substrates of transport proteins *via* fluorescence or LC-MS/MS (*e.g*.; ABC or SLCs]^41,42^, reporter assays for downstream measurement of reporter genes [*e.g*., for G-protein coupled receptors (GPCRs)^43^], membrane potential assays to assess (passive) ion flow [*e.g*., for ion channels (ICs)], or ATPase activity measurement by colorimetric detection of phosphate production [*e.g*., ABC transporters, tyrosine kinases (TKs)]^40^.

### Suggested structure-activity relationships (SARs)

**Fig. 5:**
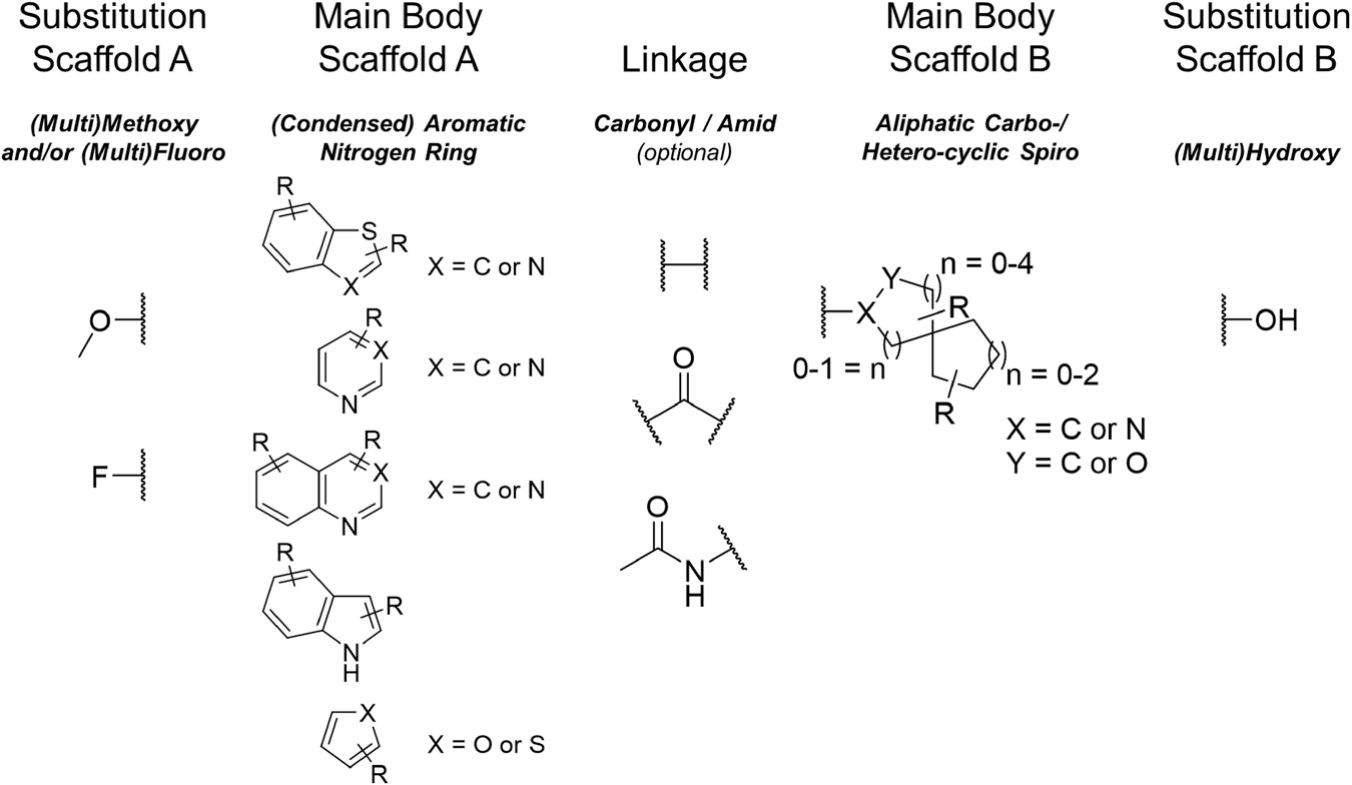
Suggested preliminary SARs and explorative, combinatorial chemistry approach to subsequently gain more, novel ABCA1 modulators as a result of the pattern scoring approach as described within the dataset and this work.

Despite the strong emphasis on molecular-structural diversity within the dataset and the presented workflow leading to the purchase of 66 molecular-structurally and physicochemically different small-molecules, the identified hit molecules, particularly **4,** **7,** **8,** **11,** **13,** **28,** **29,** **30,** **32,** **41,** **46,** **47,** **50,** **58**, and **59** showed reoccurring substructural elements that allowed for preliminary SARs: (i) basic scaffolds with nitrogen/chalcogen heterocycles [*i.e*., pyridine, (benzo)thiazole, pyrimidine, quinoline, (benzo)thiophene, indole, furan, and quinazoline] were preferred; (ii) spiro substructures, which seem to be critical for activity, particularly between 3- and 6-, 5- and 6-, 6- and 4-, 6- and 5-, as well as 7- and 5-membered (hetero)aliphatic rings containing preferably 1–2 hydroxy groups; (iii) these basic scaffolds and spiro substructures are either directly linked or bridged *via* a carbonyl or amid group; and (iv) both the basic scaffolds and other aromatic rings at the molecule extremities preferably contain (multi)methoxy- and/or (multi)fluoro-substitutions. Figure 5 visualizes these preliminary SARs, and Sheet 7, Table W of the dataset provides a list of suggested potential ABCA1 modulators for future synthetic exploration. These suggested molecules may serve other researchers to generate more potent and selective ABCA1 modulators, but also promote a new generation of ligands for other (orphan) ABCA transporters, *i.e*., ABCA2–10 and ABCA12–13.

In conclusion, the herein presented dataset and its embedded workflow demonstrated strong robustness and reliability, and moreover, were able to already elucidate preliminary SARs. This is also very surprising considering that the until today reported 14 inhibitors of ABCA1 belonged to individual molecular-structural classes without any known SARs^21^. The workflow allows medicinal chemists around the world not only to just gain a novel starting point for the exploration of orphan targets but also to provide potent molecules with an already preliminary understanding of their potential molecular interaction with the orphan target-of-interest.

### Supplementary information

C@PS_Sci_Data_Supplementary_Information_Final

## Data Availability

The entire workflow is available as dataset along with this article, and can also freely be accessed on zenodo (http://www.zenodo.org)^29^ as well as the PANABC web page (http://www.panabc.info), without restrictions. Table X of the dataset provides legends with an overview of the content. No custom code was used during this study for the curation and/or validation of the dataset.
