# Supplementary material for: Computer-aided pattern scoring – A multitarget dataset-driven workflow to predict ligands of orphan targets
Source: Sci Data. 2024 May 23;11:530. doi: 10.1038/s41597-024-03343-8 (PMC11116543; doi:10.1038/s41597-024-03343-8)
Supplement: Supplementary file 1 — C@PS_Sci_Data_Supplementary_Information_Final [file 41597_2024_3343_MOESM1_ESM.pdf]

Title

Computer-aided pattern scoring – A multitarget dataset-driven workflow to predict ligands of orphan targets

Authors

Katja Stefan[1,2], Vigneshwaran Namasivayam[2,3,*], Sven Marcel Stefan[1,2,4,*]

Affiliations

1. University of Oslo and Oslo University Hospital, Dep#artment of Pathology, Rikshospitalet, Sognsvannsveien 20, 0372 Oslo, Norway
2. University of Lübeck and University Medical Center Schleswig-Holstein, Lübeck Institute of Experimental Dermatology, Medical Systems Biology Division, Medicinal Chemistry and Systems Polypharmacology, Ratzeburger Allee 160, 23538 Lübeck, Germany
3. University of Bonn, Pharmaceutical Institute, Department of Pharmaceutical and Cellbiological Chemistry, An der Immenburg 4, 53121 Bonn, Germany
4. Medical University of Lublin, Department of Biopharmacy, Chodzki 4a, 20-093 Lublin, Poland

* corresponding authors: Sven Marcel Stefan (sven.stefan@uni-luebeck.de)
Vigneshwaran Namasivayam (vnamasiv@uni-bonn.de)

Supplementary Information

Supplementary Figure S1.. P2
Supplementary Figure S2.. P3
Supplementary Figure S3.. P4

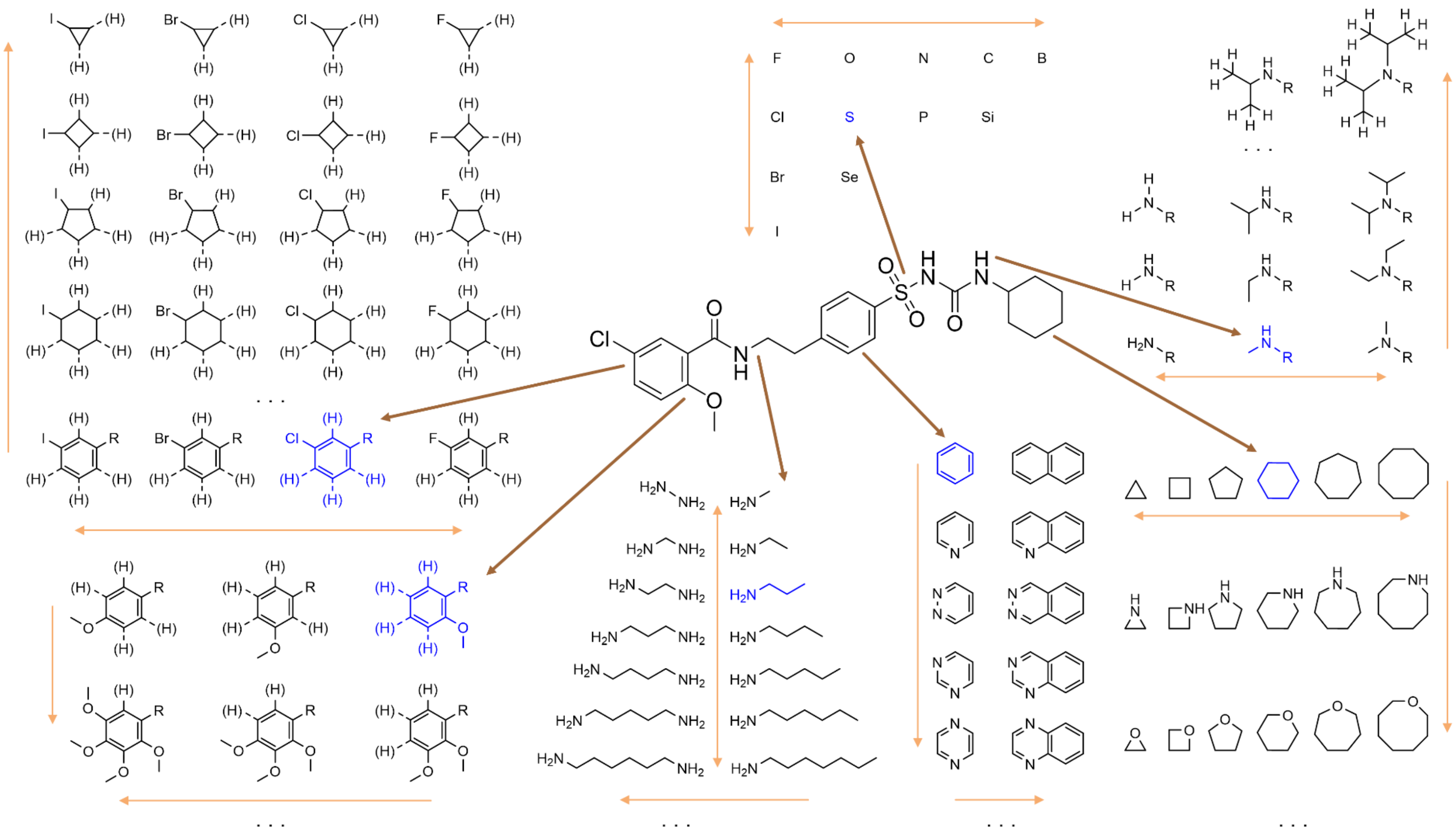

Supplementary Fig. 1 Visualization of the heavy atom distribution scheme as described earlier[1] using glibenclamide as example.

1. Namasivayam, V., Silbermann, K., Pahnke, J., Wiese, M. & Stefan, S.M. Scaffold fragmentation and substructure hopping reveal potential, robustness, and limits of computer-aided pattern analysis (C@PA). *Comput Struct Biotechnol J* **19**, 3269-3283, https://doi.org/10.1016/j.csbj.2021.05.018 (2021)

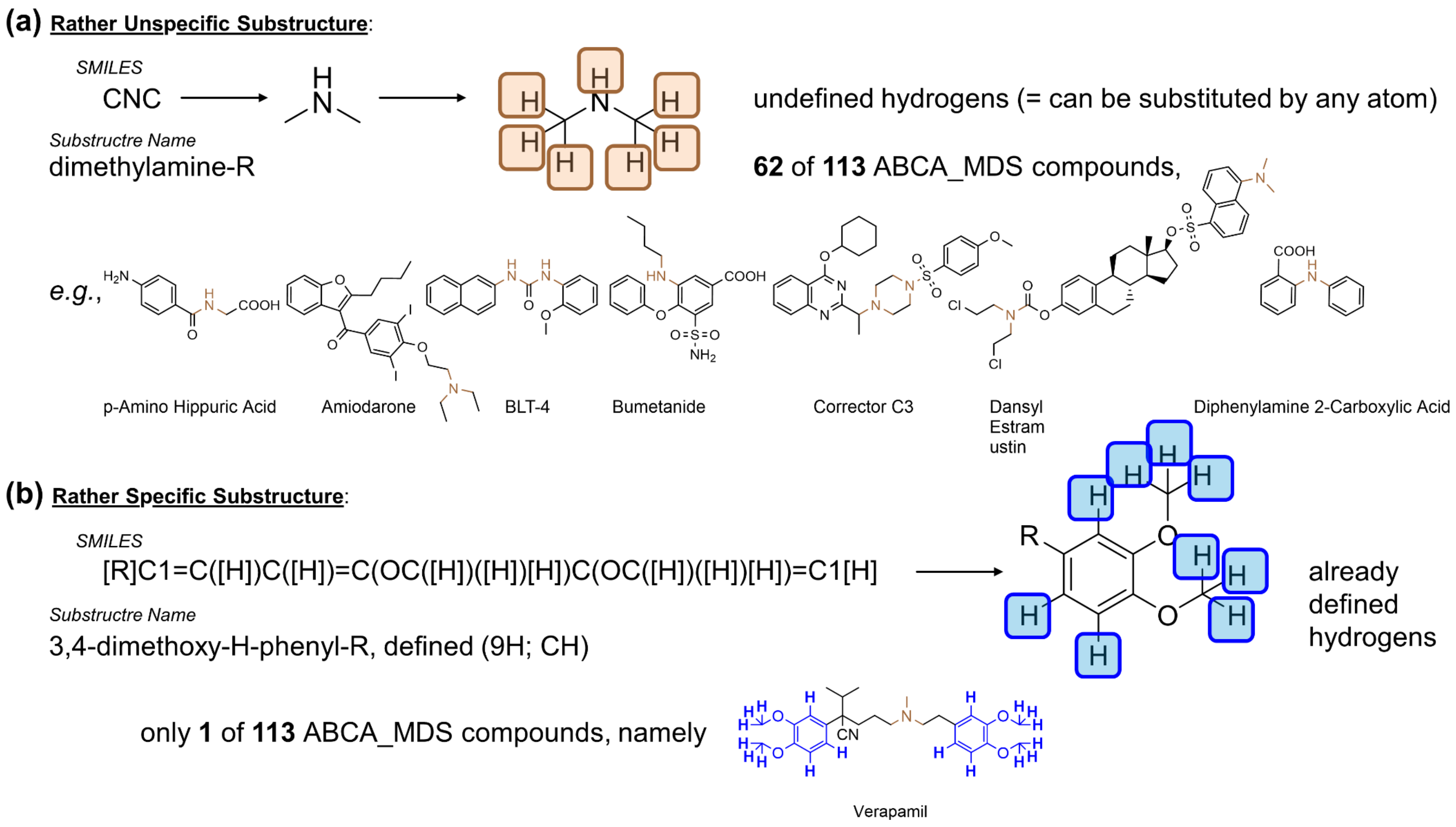

Supplementary Fig. 2 Visualization of rather unspecific (a) and rather specific (b) substructures.

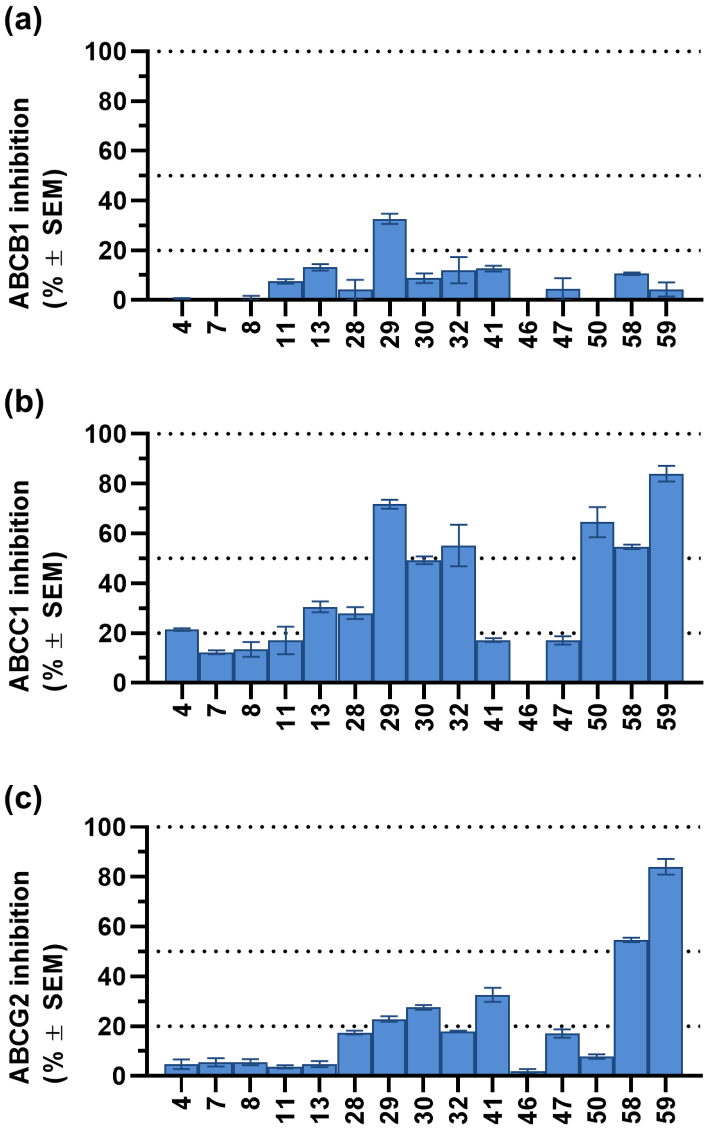

Supplementary Fig. 3 Biological Assessment of hit compounds **4**, **7**, **8**, **11**, **13**, **28**, **29**, **30**, **32**, **41**, **46**, **47**, **50**, **58**, and **59** (10 µM) against ABCB1 (a), ABCC1 (b), and ABCG2 (c) in calcein AM (a), daunorubicin (b), and pheophorbide A (c) assays using ABCB1-expressing A2780/ADR (a), ABCC1-expressing H69AR (b), and ABCG2-expressing MDCK II BCRP (c) cells as described earlier.[2]

2. Möhle, L., *et al.* ABC Transporter C1 Prevents Dimethyl Fumarate from Targeting Alzheimer's Disease. *Biology (Basel)* **12** (7), 932, https://doi.org/10.3390/biology12070932 (2023)
